# A Novel Virulence Phenotype Rapidly Assesses *Candida* Fungal Pathogenesis in Healthy and Immunocompromised *Caenorhabditis elegans* Hosts

**DOI:** 10.1128/mSphere.00697-18

**Published:** 2019-04-10

**Authors:** Dorian J. Feistel, Rema Elmostafa, Nancy Nguyen, McKenna Penley, Levi Morran, Meleah A. Hickman

**Affiliations:** a Department of Biology, Emory University, Atlanta, Georgia, USA; Carnegie Mellon University

**Keywords:** *Caenorhabditis elegans*, *Candida albicans*, host fitness, immune function, non-albicans *Candida*, virulence

## Abstract

The yeast Candida albicans is an opportunistic pathogen of humans, meaning that despite commensal interactions with its host, it can transition to a harmful pathogen. While C. albicans is the predominant species isolated in the human gastrointestinal mycobiome and is implicated in fungal infection, infections due to non-albicans *Candida* species are rapidly rising. Studying the factors that contribute to virulence is often challenging and frequently depends on many contexts, including host immune status and pathogen genetic background. Here, we utilize the nematode Caenorhabditis elegans as a perspicuous and efficient model host system to study fungal infections of *Candida* pathogens. We find that, in addition to reducing lifetime host survival, exposure to C. albicans results in delayed reproduction, which significantly reduced lineage growth over multiple generations. Furthermore, we assessed fungal pathogen virulence in C. elegans hosts compromised for innate immune function and detected increased early mortality, reduced brood sizes, and delayed reproduction relative to infected healthy hosts. Importantly, by assessing virulence in both healthy and immunocompromised host backgrounds, we reveal the pathogen potential in non-albicans *Candida* species. Taken together, we present a novel lineage growth assay to measure reduction in host fitness associated with fungal infection and demonstrate significant interactions between pathogen and host immune function that contribute to virulence.

**IMPORTANCE** Opportunistic pathogens are commensals capable of causing disease and are serious threats to human health. It is critical to understand the mechanisms and host contexts under which opportunistic pathogens become virulent. In this work, we present a novel assay to quickly and quantitatively measure pathogen virulence in healthy and immunocompromised nematode hosts. We found that *Candida* species, one of the most prominent fungal opportunistic pathogens of humans, decrease host fitness by reducing survival and impacting host reproduction. Most importantly, by measuring virulence in hosts that have intact or compromised immune function, we can reveal the pathogenic potential of opportunistic fungal pathogens.

## Abstract (precis)

Opportunistic pathogens are commensals capable of causing disease and are serious threats to human health. It is critical to understand the mechanisms and host contexts under which opportunistic pathogens become virulent. In this work, we present a novel assay to quickly and quantitatively measure pathogen virulence in healthy and immunocompromised nematode hosts. We found that *Candida* species, one of the most prominent fungal opportunistic pathogens of humans, decrease host fitness by reducing survival and impacting host reproduction. Most importantly, by measuring virulence in hosts that have intact or compromised immune function, we can reveal the pathogenic potential of opportunistic fungal pathogens.

## INTRODUCTION

*Candida* species are commensals of the human gastrointestinal microbiota and various other niches in the human body (1). Despite their commensal existence in many humans, Candida albicans accounts for more than 400,000 invasive fungal infections worldwide and is the fourth most prevalent cause of all nosocomial bloodstream infections (2–4). The severity of fungal infection is often dependent on the immune status of the host, with mucosal infections occurring in healthy individuals and bloodstream infections in immunocompromised hosts (5–9). The majority of these infections are associated with Candida albicans; however, other, non-albicans *Candida* species are increasingly becoming implicated in fungal infections (10, 11).

Several host models have been employed to better understand fungal infection and disease progression. These experimental systems include mouse, zebrafish, nematode, wax moth, and human *ex vivo* models have been specifically developed to study *Candida* infections (12). Most often, virulence phenotypes are limited to survival outcomes or tissue-specific fungal burden (13), the latter of which requires animal sacrifice and cannot address the long-term consequence of fungal disease. Furthermore, animal mortality and sacrifice, particularly in vertebrate models, constrain the number of infected individuals to study and thus reduce the quantitative robustness of these experiments. Nonetheless, these studies provide valuable insight into *Candida* infection and disease and have uncovered important roles for innate immune function of hosts (14–16), specific C. albicans genes regulating virulence (17, 18) and differences between genetic backgrounds within and between *Candida* species (19).

Other major limitations for most studies of *Candida* infection virulence include the time-intensive nature of tracking virulence phenotypes in vertebrate systems such as mice, the genetic intractability of zebrafish and wax moth larvae and the context-independent nature of *ex vivo* models (5). As such, most studies focus specifically on C. albicans virulence, while many non-albicans *Candida* species are evaluated to a lesser degree. The nematode Caenorhabditis elegans has been developed as a model for *Candida* infection to overcome these limitations (20, 21) and to screen compounds for antifungal activity (22). C. elegans has proven useful for studying host-microbe interactions (23) because many fungal and bacterial pathogens that cause illness in humans also cause disease in C. elegans (24). Infecting C. elegans is relatively simple and often done by replacing or incorporating its standard laboratory food E. coli with a desired pathogen which colonizes the gut and causes disease (24). Furthermore, the innate immune system in C. elegans includes SEK-1, a MAP kinase kinase (MAPKK) that is homologous to the MKK3/6 and MKK4 family of mammalian MAPKKs and activates the C. elegans p38 MAP kinase ortholog (25). This pathway has been suggested to be an ancient and conserved component of C. elegans’ immune response to pathogens (26). As such, the *sek-1* mutation increases susceptibility to microbial colonization (27), including infection with many *Candida* species (28) and is necessary to induce the appropriate antifungal immune defense (29).

In addition to utilizing innate immune system mutants, most studies measure host mortality in temperature-sensitive sterile mutants (18, 22, 30, 31) to more easily track mortality in founder populations since C. elegans has a rapid life cycle and large brood sizes (32, 33). The effects on host reproduction are often overlooked in favor of examination of mortality rates when assessing a pathogen’s virulence (34). Yet, it is important to remember that virulence can be broadly measured as any reduction in host fitness resulting from interactions between a pathogen and its host (35–37). In this work, we assessed virulence of *Candida* species in C. elegans hosts using a novel measure of host fitness that incorporated both host survival and fecundity. We found that fungal pathogens reduced host fitness by delaying reproduction, resulting in long-term consequences for population growth in both healthy and immunocompromised host backgrounds. Using this novel measure, we characterized virulence phenotypes for three non-albicans *Candida* species—C. dubliniensis, C. tropicalis, and C. parapsilosis—in both healthy and immunocompromised C. elegans hosts. Our studies demonstrate that differences in virulence can be identified between pathogen species and that pathogenic potential is often revealed when host immune function is diminished.

## RESULTS

### Exposure to *C. albicans* reduces multiple measures of *C. elegans* fitness.

**FIG 1:**
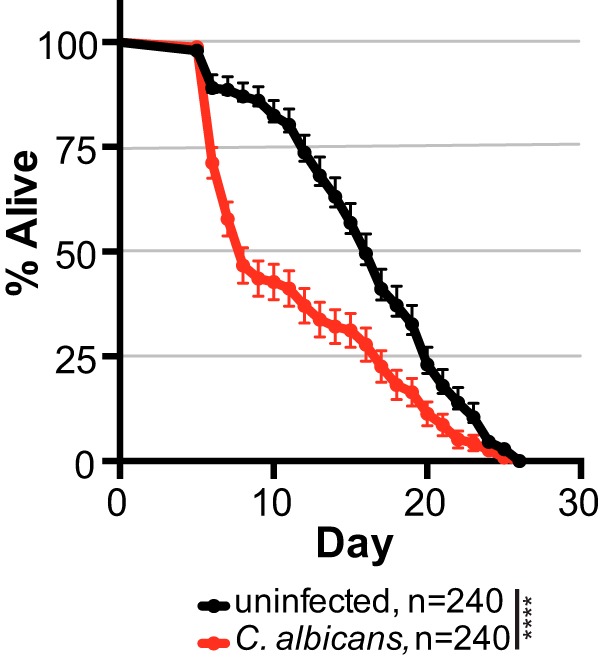
Reduced nematode survival associated with C. albicans infection. Survival curves for C. elegans populations that are either uninfected (exposed to just an E. coli food source, black) or when infected with C. albicans SC5314 (red). Mean data values are plotted with bars showing the standard errors of the mean (SEM). The number of worms analyzed (*n*) for each treatment is indicated. ****, *P* < 0.0001 (log-rank [Mantel-Cox] test).

Pathogens affect host fitness by decreasing life span and/or reducing fecundity. To determine how C. albicans impacts C. elegans host fitness, we first measured nematode life span in the presence or absence of C. albicans. We observed a significant reduction in survival when C. elegans was reared on C. albicans compared to when it was reared in its absence (Fig. 1, *P* < 0.0001 [log-rank test]), consistent with previously published results (38). A 50% mortality was reached in 8 days in nematode populations exposed to C. albicans compared to 16 days in unexposed nematode populations, indicating that C. elegans lifespan is substantially decreased by exposure to C. albicans.

**FIG 2:**
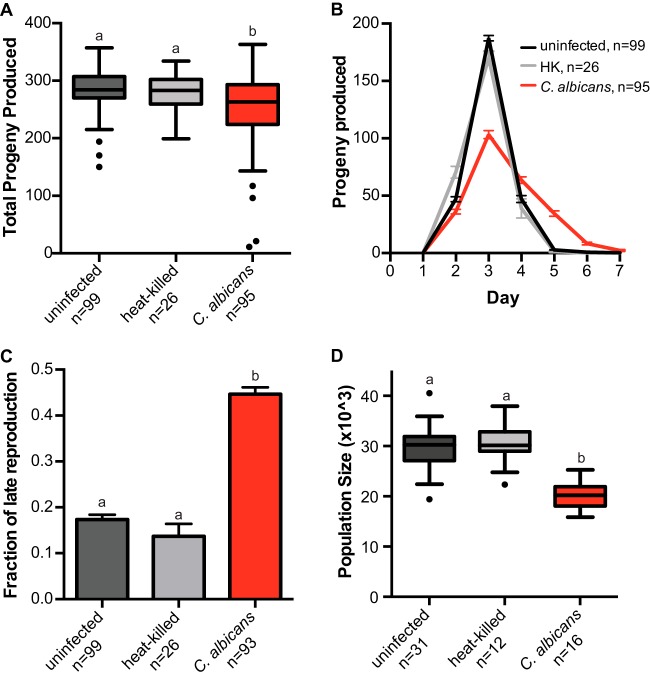
Live C. albicans reduces nematode fecundity. (A) Box-and-whiskers plot of brood sizes for C. elegans (N2) exposed to an E. coli food source alone (uninfected, black), heat-killed C. albicans (gray), or live C. albicans (red). Boxes show the 25th to 75th quartiles, with the medians indicated. Error bars are the normalized range of the data, and circles indicate outliers. (B) Number of viable C. elegans progeny produced per day in uninfected, heat-killed, and live C. albicans treatments. Data represent means and SEM. (C) Fraction of C. elegans progeny produced on day 4 or later during the “late reproductive” window. Data represent the means and SEM. (D) Box-and-whiskers plot of average population size (representing the number of F_1_ and F_2_ progeny) produced within 7 days from a single founder C. elegans. Boxes indicate the 25th to 75th quartiles, with the medians indicated. Error bars indicate the normalized range of the data, and circles indicate outliers. The number (*n*) of experimental samples analyzed is indicated for each treatment. Treatments that share letters are not significantly different, whereas treatments with different letters are spastically different according to a *post hoc* Dunn’s multiple-comparison test.

While overall survival was reduced, nematode death was rarely observed earlier than 5 days postinfection. Importantly, adult nematodes produce the majority of their offspring before C. albicans infection reduces rates of survival. Given this information and that fecundity is a key component of fitness from an evolutionary perspective, we investigated whether C. albicans negatively impacts C. elegans reproduction. To test this, we counted the total number of viable progeny (i.e., brood size) produced in the first 7 days of reproductive maturity from individual nematodes that were either unexposed or exposed to either heat-killed or live C. albicans. As shown in Fig. 2A, exposure to C. albicans reduced the average number of viable progeny (253 ± 7) compared to the unexposed control (285 ± 4; *P < *0.0002 [Kruskal-Wallis test]) by ∼11%. While statistically significant, the cost of C. albicans toward host brood size is modest. Furthermore, the brood size of nematodes exposed to heat-killed C. albicans (HK) was not detectably different from uninfected or live C. albicans treatments (Fig. 2A).

Intriguingly, we identified a significant delay in reproductive timing when we measured the number of progeny produced every 24 h during the host reproductive window. We observed a substantial reduction in reproduction on day 3 in nematodes exposed to C. albicans (103 ± 4) relative to unexposed hosts (187 ± 3) or hosts associated with heat-killed C. albicans (Fig. 2B; *P < *0.0001 [Kruskal-Wallis test]) and increased progeny produced on Days 4–6 in nematodes exposed to C. albicans (Fig. 2B). We calculated the total fraction of reproduction occurring in this “late” window (days 4 to 6) and identified a significant increase in the progeny produced late in C. elegans exposed to C. albicans compared to unexposed or heat-killed controls (Fig. 2C; *P = *0.0001 [Kruskal-Wallis test]). Taken together, our data indicate that C. albicans severely delays and reduces reproduction, in addition to impacting overall survival in C. elegans.

To investigate whether this reproductive delay observed in nematodes exposed to C. albicans had any long-term consequences, we took advantage of the fact that C. elegans are hermaphrodites and measured the total population produced from a single founder worm over multiple generations through lineage expansion assays. We find that the median population produced within 7 days from a single uninfected nematode is ∼30,250 F_1_ and F_2_ progeny (Fig. 2D). Lineage expansion is significantly impacted in nematodes exposed to live C. albicans, with a median population size of ∼20,208 F_1_ and F_2_ progeny, a reduction of ∼33% compared to uninfected and heat-killed control treatments (Fig. 2D and *P* < 0.0001, determined using one-way analysis of variance [ANOVA]). We conclude that the delay in reproduction, coupled with the reduced brood size resulting from exposure to C. albicans, severely impacts C. elegans evolutionary fitness.

**FIG 3:**
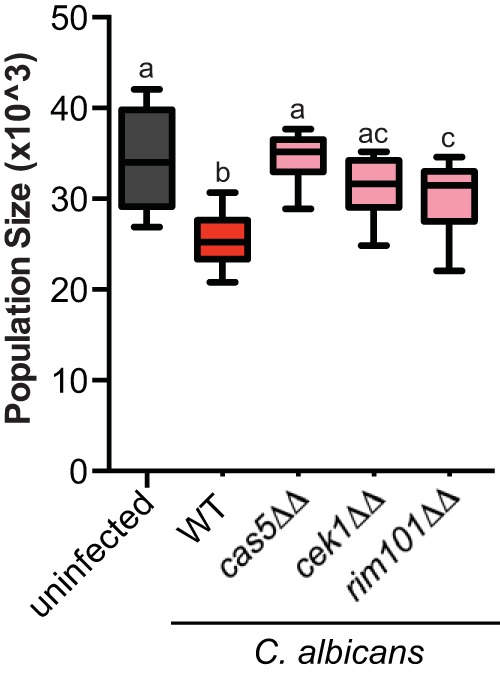
C. albicans deletion strains are avirulent or have attenuated virulence. A box-and-whiskers plot shows the average population size (representing the number of F_1_ and F_2_ progeny) produced within 7 days from a single founder, C. elegans (N2), exposed to an E. coli food source alone (uninfected *n* = 10, black), wild-type (WT) C. albicans (SN250, *n* = 12, red), or C. albicans *cas5ΔΔ* (*n* = 12), *cek1ΔΔ* (*n* = 12), and *rim101ΔΔ* (*n* = 12) mutant strains (pink). Boxes indicate the 25th to 75th quartiles, with the medians indicated. Error bars indicate the normalized range of the data. Treatments that share letters are not significantly different, whereas treatments with different letters are statistically different, according to a *post hoc* Dunn’s multiple-comparison test.

To address whether the decreases in host fitness we observed are due to the pathogenicity of C. albicans or simply the result of exposure to live fungal cells, we measured C. elegans lineage expansion with several homozygous mutants of C. albicans with reduced virulence or avirulent phenotypes previously identified. We tested deletions of *CAS5*, a transcription factor that regulates cell wall homeostasis, cell adhesion, and stress response (18, 39); *CEK1*, a kinase required for the yeast-hypha transition (40–42); and *RIM101*, a transcription factor involved in the alkaline pH response (18, 43–45). We detect significant differences between host population sizes among these treatments (*P < *0.0001 [one-way ANOVA]). For all three C. albicans deletion strain infections, host lineage growth was significantly larger than lineage growth associated with wild-type C. albicans (Fig. 3). Moreover, infections with *cas5ΔΔ* and *cek1ΔΔ* strains resulted in lineage growth that is indistinguishable from the uninfected control (Fig. 3), indicating that these two mutants are avirulent in our C. elegans infection model. Deletion of *RIM101* has an attenuated virulence phenotype and displays a significant reduction in host lineage growth compared to the uninfected control, but not to the same severity as wild-type C. albicans (Fig. 3). Taken together, these results indicate that reduction in C. elegans host fitness is not a general response to foreign fungal cells but results from the pathogenesis of C. albicans.

### Immunocompromised hosts are susceptible to fungal infection.

**FIG 4:**
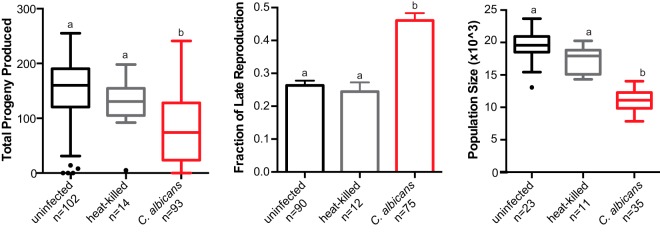
C. albicans severely impacts host fitness in immunocompromised nematodes. (Left) Box-and-whiskers plot of brood sizes for C. elegans (N2) exposure to E. coli food source alone (uninfected, black), heat-killed C. albicans (gray), or live C. albicans (red). Boxes indicate the 25th to 75th quartiles, with the medians indicated. Error bars indicate the normalized range of the data and circles indicate outliers. (Middle) Fraction of C. elegans progeny produced on day 4 or later during the “late reproductive” window. Data represent the means and the SEM. (Right) Box-and-whiskers plot of the average population size (representing the number of F_1_ and F_2_ progeny) produced within 7 days from a single founder C. elegans. Boxes indicate the 25th to 75th quartiles, with the medians indicated. Error bars indicate the normalized range of the data, and circles indicate outliers. The number (*n*) of experimental samples analyzed is indicated for each treatment. Statistical significance is indicated when treatments differ. Treatments that share letters are not significantly different, whereas treatments with differing letters are statistically different, according to *post hoc* Dunn’s multiple-comparison tests.

A leading host-related risk factor for invasive candidiasis is compromised immune function (46). Given our novel results regarding fecundity in healthy C. elegans hosts, we were curious how exposure to C. albicans impacts nematode hosts with compromised immune function. To address this point, we utilized a C. elegans strain carrying a mutation in SEK-1, a well-conserved MAP kinase involved in the innate immune signaling cascade (25, 27) and antifungal response (29). Unlike healthy hosts, immunocompromised hosts show high rates of early (within 7 days) mortality in both uninfected and C. albicans treatments (Fig. S1). C. albicans exposure reduced average brood size by nearly 50% (78 ± 6) in immunocompromised hosts compared to uninfected (151 ± 5) and heat-killed (127 ± 13) controls (Fig. 4, left panel , and *P < *0.0001 [Kruskal-Wallis test]). It is important to note that in all treatments (unexposed, heat-killed, and live C. albicans), immunocompromised hosts produced significantly less progeny than healthy hosts (see Table S1 in the supplemental material), but the largest reductions occur in immunocompromised hosts are exposed to live C. albicans.

10.1128/mSphere.00697-18.1 FIG S1 Immunocompromised hosts survive less well than healthy hosts. Mean survival of healthy N2 (solid lines) and immunocompromised *sek-1* (dashed lines) C. elegans populations that are either uninfected (black) or when infected with C. albicans SC5314 (red). Mean data values are plotted with SEM error bars; the number of worms analyzed (n) for each treatment is indicated in the legend. Download FIG S1, EPS file, 0.7 MB. Copyright © 2019 Feistel et al. 2019 Feistel et al. This content is distributed under the terms of the Creative Commons Attribution 4.0 International license.

10.1128/mSphere.00697-18.4 TABLE S1 Brood size, fraction of late reproduction, and lineage expansion population size of healthy (N2) and immunocompromised (*sek*-*1*) hosts when treated with food alone (uninfected), heat-killed (MH88), or live *Candida* species. The data are the average ± the SEM, and the total number of worms analyzed (n) is provided. *P* values are given for two-tailed *t* tests comparing healthy and immunocompromised individuals for each treatment. Download Table S1, PDF file, 0.07 MB. Copyright © 2019 Feistel et al. 2019 Feistel et al. This content is distributed under the terms of the Creative Commons Attribution 4.0 International license.

One explanation for the overall reduction in average brood size is the increased early mortality we observe in immunocompromised hosts. To address this possibility, we reanalyzed brood size only from hosts that survived past day 3 and found that while this did slightly increase average brood size across all treatments, it was not significant between analyses (*P* = 0.2356 [two-tailed *t* test]), and exposure to C. albicans still significantly reduced average brood size (94 ± 6) relative to uninfected (163 ± 5) and heat-killed (141 ± 9) controls (Fig. S2, *P < *0.0001 [Kruskal-Wallis test]). For hosts that survived past 3 days, we also detected a significant delay in reproductive timing upon exposure to C. albicans, with nearly 45% of all reproduction occurring in this late window (Fig. 4, middle panel), similar to what we observed in healthy hosts (Table S1).

10.1128/mSphere.00697-18.2 FIG S2 Reduced brood size in C. albicans-infected immunocompromised hosts is not due to early death. A box-and-whiskers plot of brood sizes for immunocompromised C. elegans (*sek-1*) that are still alive on or by day 4 that have been exposed to E. coli food source alone (uninfected, black) or live C. albicans (red) is shown. Boxes indicate the 25th to 75th quartiles, with the medians indicated. Error bars are the normalized range of the data, and circles indicate outliers. The number (n) of experimental samples analyzed is indicated for each treatment. Treatments that share letters are not significantly different, whereas treatments with differing letters are statistically different, according to a *post hoc* Dunn’s multiple-comparison test. Download FIG S2, EPS file, 0.7 MB. Copyright © 2019 Feistel et al. 2019 Feistel et al. This content is distributed under the terms of the Creative Commons Attribution 4.0 International license.

**FIG 5:**
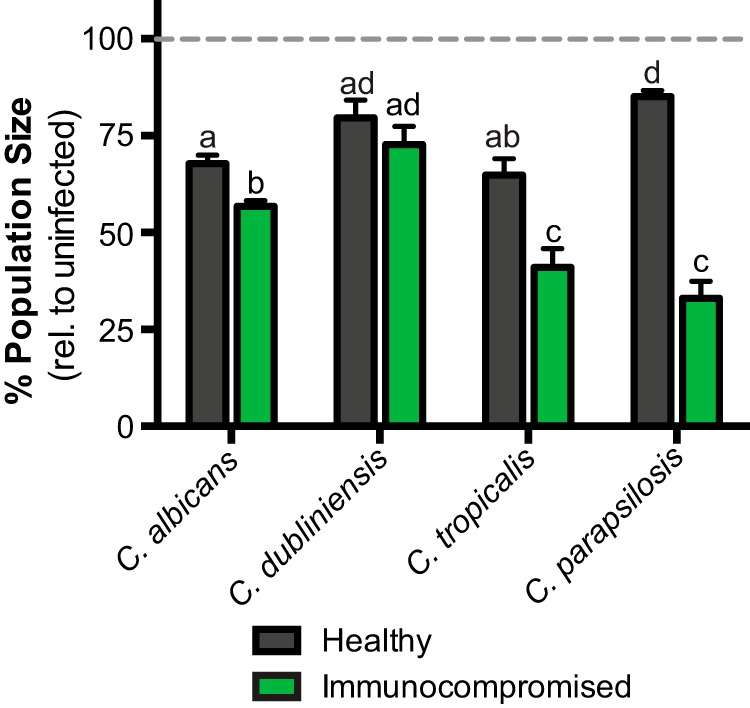
Virulence of *Candida* species depends on host immune function. Reductions in relative population size for healthy (N2, black bars) and immunocompromised (*sek-1*, green bars) hosts exposed to C. albicans, C. dubliniensis, C. tropicalis, and C. parapsilosis are shown. Dashed line indicates the relative population size of uninfected hosts. Interactions between *Candida* species and host background were determined by two-way ANOVA (*P* < 0.001). Treatments that share letters are not significantly different, whereas treatments with different letters are statistically different, according to a *post hoc* Tukey’s multiple-comparison test. The number (*n*) of experimental samples analyzed is indicated for each treatment in Table S1.

We predicted that lineage expansion in immunocompromised hosts exposed to C. albicans would be more dramatically impacted relative to wild-type nematodes exposed to C. albicans, given the reproductive delays coupled with the overall reduced brood sizes and increased mortality observed. Indeed, we determined that the median population size after 7 days of C. albicans exposed nematodes was ∼11,000 compared to ∼17,000 and 19,500 in heat-killed and uninfected controls, respectively (Fig. 4, right panel). The overall reduction in population size resulting from C. albicans exposure is ∼43% in immunocompromised hosts and is significantly greater than the ∼32% reduction in population size in healthy hosts (Fig. 5 and Table S1). Taken together, our results indicate that immunocompromised hosts are more susceptible to fungal infection than healthy wild-type hosts.

### Host immune status reveals pathogenic potential in other *Candida* species.

While C. albicans is the predominant fungal agent in invasive candidiasis, non-albicans *Candida* species are estimated to account for 35 to 65% of candidemias (47). By evaluating non-albicans *Candida* species in both healthy and immunocompromised hosts, we can begin to assess their pathogenic potential and thus extend the utility of C. elegans as a model host system for detecting virulence in *Candida* species. Here, we leveraged our simple, yet robust, lineage expansion assays to measure the impact of three additional *Candida* species—C. dubliniensis, C. tropicalis, and C. parapsilosis—on host fitness for healthy and immunocompromised C. elegans. All three non-albicans *Candida* species significantly reduced the population sizes of both healthy and immunocompromised hosts compared to uninfected controls (black asterisks, Fig. S3 and Table S1) and sometimes were also significantly different than the population sizes of hosts exposed to C. albicans (red asterisks, Fig. S3).

10.1128/mSphere.00697-18.3 FIG S3 Non-albicans *Candida* species differentially impact host fitness depending on host immune status. A box-and-whiskers plot shows the average population size from lineage expansion assays (representing the number of F_1_ and F_2_ progeny) produced within 7 days from a single founder C. elegans for healthy hosts (N2) (A) or immunocompromised hosts (*sek-1*) (B) exposed to C. dubliniensis, C. tropicalis, and C. parapsilosis. Boxes indicate the 25th to 75th quartiles with the medians indicated. Error bars indicate the normalized range of the data, and circles indicate outliers. The number (*n*) of experimental samples analyzed is indicated for each treatment. Treatments that share letters are not significantly different, whereas treatments with differing letters are statistically different, according to a *post hoc* Dunn’s multiple comparisons test. The mean population size for uninfected (black line) and C. albicans exposed (red line) hosts is indicated for reference. Asterisks indicate significant differences from uninfected (black) or live C. albicans (red) for each non-albicans *Candida* species (*post hoc* Dunn’s multiple-comparison tests). Download FIG S3, EPS file, 0.7 MB. Copyright © 2019 Feistel et al. 2019 Feistel et al. This content is distributed under the terms of the Creative Commons Attribution 4.0 International license.

We next wanted to determine whether there was a significant interaction between *Candida* species and host immune function. To test this, we evaluated the population size of infected nematodes relative to uninfected for healthy (Fig. 5, black bars) and immunocompromised (Fig. 5, green bars) host backgrounds, since immunocompromised hosts have significantly smaller populations even when uninfected (Fig. 4 and Fig. S2 ). There are significant differences among *Candida* species and nematode host backgrounds (*P* < 0.001 [two-way ANOVA]; *P* < 0.001 between pathogens, hosts, and their interaction). In healthy hosts, C. albicans and C. tropicalis are the most virulent, with 32 to 35% reductions in total population size, whereas C. parapsilosis and C. dubliniensis result in more modest population reductions (17 to 20%). However, in immunocompromised hosts, C. parapsilosis is one of the most virulent, with nearly a 67% reduction in population size. C. albicans and C. tropicalis are also highly virulent in immunocompromised hosts and all three of these species more severely impact immunocompromised hosts compared to healthy hosts. In contrast, C. dubliniensis is the least virulent, with no significant differences observed between the relative population sizes of healthy and immunocompromised hosts (Fig. 5). Given the virulence phenotypes of these different yeasts, our results suggest that fungal virulence depends not only on the species of pathogen but also on the immune status of the host.

## DISCUSSION

Here we utilized a C. elegans experimental host system to identify novel measures of host fitness associated with fungal infection. By tracking the number of progeny produced per day, we can quickly and quantitatively assess three aspects of host fitness: early mortality, total viable offspring produced, and reproductive timing. We show that exposure to Candida albicans in healthy Caenorhabditis elegans hosts not only reduces survival (Fig. 1) but modestly reduces total progeny produced and dramatically delays reproductive timing (Fig. 2). This reproductive delay has long-term consequences for lineage expansion, since single founder nematodes exposed to C. albicans reduce its population growth by ∼30% (Fig. 2D). Furthermore, reductions in host population growth are dependent on C. albicans virulence rather than a general host response to foreign fungal cells since host population growth was minimally affected in C. albicans virulence deletion strains (Fig. 3). We found similar delays in reproductive timing in immunocompromised hosts exposed to C. albicans, although immunocompromised hosts are more susceptible to infection and have higher incidence of early mortality and smaller brood sizes compared to healthy hosts (Fig. 4). Importantly, the delayed reproduction phenotype in infected C. elegans is easily ascertained, highly quantitative, and reproducible and can be used as a screening method to detect relatively small differences in virulence across *Candida* strains or other fungal species. Furthermore, by utilizing different host contexts, this assay revealed the pathogenic potential of other important, yet understudied, *Candida* species (Fig. 5), including C. parapsilosis, which has dramatically higher levels of virulence in immunocompromised hosts compared to healthy hosts.

The reductions in host fitness associated with C. albicans and other *Candida* species indicate that we have established a *bona fide* model of fungal infection. Importantly, these reductions in host fitness cannot be attributed to *Candida* species being a suboptimal food source or that the hosts are starving because we deliver the pathogen with E. coli, the standard food source for C. elegans. Further, we do not detect any host larval development into dauer, an alternative life stage in response to starvation and overpopulation (33). While pathogen avoidance is a common defense strategy for C. elegans (48), we censored any worms that have crawled off the plate or have disappeared during the course of our experiment and removed them from our data analysis. Microscopic analysis reveals that fungal cells inhabit the gut of C. elegans (38; data not shown), and we can extract viable host-associated fungal cells (data not shown). Furthermore, the reductions in host fitness depend on ingesting live C. albicans, since heat-killed treatments do not cause significant reductions in total brood size, reproductive timing, or lineage expansion (Fig. 2, HK), and deletion of previously identified virulence genes (18, 39, 41, 43) resulted in avirulent or attenuated virulence phenotypes (Fig. 3).

The host reproductive delay we observed in C. elegans upon fungal infection is a robust, highly quantitative measure of virulence that makes it amenable to screen a variety of host and pathogen genetic backgrounds that has been challenging in mammalian and insect models. C. elegans is easily maintained in the lab, has a short life cycle that generates a large number of progeny, and has been a fundamental model genetic organism (33). Previous work has shown that upon exposure to C. albicans, C. elegans have a transcriptional response consistent with that of infection (29). In the present study, we utilized a mutant C. elegans strain in which *SEK-1*, an important MAPK in innate immunity and whose homologs include the MKK3/6 and MKK4 family of mammalian MAPKKs, to determine the severity of fungal infection in immunocompromised hosts. We demonstrate that deficiencies in innate immune function result in hosts with high susceptibility to fungal infection and in the future can extend this analysis for many other host backgrounds that are mutant for immune function.

Previous studies using C. elegans as a host for fungal infection have described additional phenotypes beyond reduced survival, including a deformed anal region (DAR) (38, 49). It is possible that DAR may be contributing to the delayed reproductive phenotype we observe in our experiments; however, the frequency of DAR phenotypes was low, and we did not observe any detectable differences between hosts exposed to C. albicans and hosts unexposed (data not shown), nor did we observe dramatic reductions in total number of viable offspring produced between these two groups of hosts (Fig. 2A). While this deformity is a result of a local defense reaction of the worm due to extracellular signal-regulated kinase activation of the innate immunity MAKK cascade, it is a distinct marker from delayed reproduction for infection (50). Normal reproductive timing utilizes the highly conserved DAF-2/DAF-16 insulin-like signaling pathway (24). This signaling pathway has also been implicated in innate immunity and bacterial infection (51) and is potentially disrupted in C. elegans hosts infected with *Candida*.

Impacts on host reproduction have often been overlooked in studies regarding *Candida* virulence, yet host reproduction is an important evolutionary measure of an organism’s fitness. Not only can we utilize these fecundity assays to assess the roles of host immune function on fungal pathogenesis, we can also readily screen diverse pathogen strain backgrounds and species. A large-scale, international 10-year study identified 31 species of *Candida* associated with clinical samples. While C. albicans is still the most prevalent, its isolation from clinical isolates is decreasing with corresponding increases isolation of C. glabrata, C. tropicalis, and C. parapsilosis (52). Despite its close evolutionary relationship with C. albicans, C. dubliniensis does not seem to be highly pathogenic (53). However, other non-albicans *Candida* species have been increasingly implicated in fungal infections of humans (54). For example, C. tropicalis and C. parapsilosis are common fungal species isolated in patients with candidemia (7 to 48% and 11 to 27%, respectively, depending on the geographic region), candiduria (8 to 44% and 0.5 to 11%, respectively), and oral candidosis (5 to 13% and 7 to 15%, respectively) (see reference 54 and references therein). Despite the increasing incidence of non-albicans *Candida* infections (52), experimental studies using these pathogens remains limited. Here, we analyzed the virulence phenotypes of three non-albicans *Candida* species—C. dubliniensis, C. tropicalis, and C. parapsilosis—in both healthy and immunocompromised host backgrounds. In healthy C. elegans hosts, C. parapsilosis is the least virulent compared to C. albicans and C. tropicalis and C. dubliniensis has intermediate level of virulence (Fig. 5). For all species except for C. dubliniensis, virulence is more severe in immunocompromised hosts and is particularly striking in C. parapsilosis, which is the most virulent in hosts with compromised immune function. Therefore, the C. elegans fecundity assays we developed in this study can rapidly reveal pathogenic potential by assessing virulence in multiple host genetic backgrounds and across pathogens in a highly quantitative and robust manner.

## MATERIALS AND METHODS

### Strains and media.

For this study, the fungal pathogens C. albicans (SC5314 [55] and SN250 [56]) and the C. albicans homozygous *cas5ΔΔ*, *cek1ΔΔ*, and *rim101ΔΔ* deletion strains (56), as well as C. dubliniensis (Wu284 [57]), C. tropicalis (ATCC 22109), and C. parapsilosis (ATCC 22109) strains, were used. C. elegans N2 Bristol (WT) and a *sek-1* mutant derivative (25) were used to test host survival, fecundity, and population growth. C. elegans populations were maintained at 20°C on 100 mm petri dishes with 25 ml of lite nematode growth medium (NGM; US Biological) with Escherichia coli OP50 as a food source. Nematodes were transferred to a newly seeded E. coli plate every 3 to 4 days. For survival, fecundity, and population growth assays, NGM was supplemented with 0.08 g/liter uridine, 0.08 g/liter histidine, and 0.04 g/liter adenine to facilitate growth of auxotrophic C. albicans strains and 0.2 g/liter streptomycin sulfate to inhibit E. coli overgrowth so fungal strains could proliferate.

### Seeding NGM plates for survival, fecundity, and lineage growth assays.

*Candida* strains and E. coli OP50 strains were inoculated in 3 ml of yeast extract-peptone-dextrose (YPD) or 5 ml of Luria-Bertani (LB) medium, respectively, and cultured at 30°C for 1 to 2 days. *Candida* culture densities were measured with a spectrophotometer and diluted to a final volume of 3.0 OD_600_ per ml (∼6 × 10^7^ cells per ml). E. coli cultures were pelleted and washed twice with 1 ml of double-distilled H_2_O (ddH_2_O). The supernatant was removed, and the pellet was centrifuged for 60 s at maximum to remove any excess liquid. The pellet was weighed and suspended with sterilized water to a final volume of 200 mg/ml. A mixture of 6.25 μl of E. coli and 1.25 μl of *Candida* was brought to a final volume of 50 μl with ddH_2_O. The entire 50 μl was spotted onto the center of a 35-mm-diameter supplemented-NGM Lite agar plate, followed by incubation at room temperature overnight before the addition of eggs or transferring nematodes. E. coli OP50 was used as a control at the concentration specified above.

### Egg preparation and synchronization for survival, fecundity, and population growth assays.

For survival, fecundity, and population growth assays, approximately 100 nematodes at the L3/L4 stage were transferred to a 100-mm-diameter NGM plate seeded with E. coli OP50 and maintained at 20°C for 2 to 3 days prior to the start of an experiment. On the first day of an experiment, these NGM plates were washed with M9 buffer, and the contents (live nematodes and eggs) were transferred to a 15-ml conical tube and pelleted by centrifugation (2 min at 1,200 rpm). The pellet was resuspended in a 1:4 bleach (5.25%) solution and transferred to a microcentrifuge tube. The suspension was mixed via inversion for 90 to 120 s and subsequently centrifuged (30 s at 1500 rpm). The pellet was washed with 1 ml of M9 buffer three consecutive times to remove excess bleach solution and brought to a final suspension with 500 μl of M9 buffer. To determine the concentration of eggs, 10 μl was placed on a concave slide, the eggs were counted, and the egg suspension was diluted with M9 to a final concentration of 10 eggs/μl. All assays were treated equally on the first day (day 0) by adding roughly 100 eggs to a treatment or E. coli plate (described above).

### Survival assays.

This experimental procedure is a modified version of a previously published method (38). Briefly, 72 h after adding ca. 50 to 75 nematode eggs to a plate, 40 adult nematodes were randomly selected and transferred to newly seeded plates using the concentration of food described above and then incubated at 20°C. Every other day, nematodes were transferred to freshly seeded plates until all nematode replicate populations went extinct. Occasionally, a nematode would crawl off the treatment plate or was otherwise unaccounted for and would be marked as censored. The numbers of living, dead, and censored worms were scored daily. Each survival assay was performed at least three independent times.

### Fecundity assays.

For both fecundity and lineage expansion assays, 48 h after the nematode eggs were added to a plate, a single L4 reproductively immature hermaphroditic nematode was randomly selected and transferred (6 to 10 independent biological replicates per treatment per block) to a newly seeded 35-mm petri plate containing a treatment of either C. albicans plus E. coli or E. coli alone (described above), followed by incubation at 20°C. Nematodes were transferred to freshly seeded plates every 24 h for seven consecutive days. Eggs remained undisturbed on the plate and were incubated at 20°C for an additional 24 h to provide enough time for the eggs to hatch, at which point the numbers of viable progeny per day were scored. Nematodes that died during the assay were scored as dead at the time of transfer. Nematodes that crawled off the plate or were otherwise unaccountable for were considered censored and excluded from the analysis.

### Lineage expansion assay.

A single L4 reproductively immature hermaphroditic nematode was randomly selected and transferred (six biological replicates per treatment) to a freshly seeded 100-mm treatment or E. coli plate (described above) containing a 6-fold increase in food. Five days later, each plate was washed with M9 buffer until the majority of the nematodes were displaced and subsequently transferred to 15-ml conical tubes. Tubes were placed at 4°C for 1 h to allow the nematodes to settle at the bottom. All tubes were concentrated to a final volume of 10 ml. Six 20-μl samples were taken from each population and counted.

### Statistical analyses.

All statistical analyses were performed with GraphPad Prism. Data sets were tested for normality using the D’Agostino and Pearson omnibus normality test. For comparisons across groups, one-way ANOVAs and *post hoc* Tukey multiple-comparison tests or Kruskal-Wallis and Dunn’s multiple-comparison tests were performed.
