# Supplementary material for: A Novel Virulence Phenotype Rapidly Assesses Candida Fungal Pathogenesis in Healthy and Immunocompromised Caenorhabditis elegans Hosts
Source: mSphere. 2019 Apr 10;4(2):e00697-18. doi: 10.1128/mSphere.00697-18 (PMC6458437; doi:10.1128/mSphere.00697-18)
Supplement: TABLE S1 [file mSphere.00697-18-st001.pdf]

		uninfected	heat-killed *C. albicans*	Live *C. albicans*	*C. dubliniensis*	*C. tropicalis*	*C. parapsilosis*
Brood Size	Healthy (N2)	285±4 (n=99)	281±6 (n=26)	253±7 (n=95)	276±17 (n=19)	260±5 (n=9)	224±9 (n=18)
	Immunocompromised (sek-1)	151±6 (n=102)	127±13 (n=14)	78±6 (n=93)	94±17 (n=16)	105±16 (n=15)	38±13 (n=14)
	p-value	****	****	****	****	****	****
% Late Reproduction	Healthy (N2)	19±1.2 (n=99)	14±2.7 (n=26)	44±1.7 (n=93)	21±3.7 (n=19)	32±7.2 (n=9)	72±13 (n=8)
	Immunocompromised (sek-1)	26±1.5 (n=90)	24±2.8 (n=12)	46±2.2 (n=75)	40±4.7 (n=13)	30±3.4 (n=6)	30±3.4 (n=6)
	p-value	***	0.042	0.355	**	0.758	****
Lineage Expansion	Healthy (N2)	29481±756 (n=31)	30319±1181 (n=12)	19964±636 (n=16)	23492±1358 (n=15)	19115±1271 (n=16)	24437±799 (n=9)
	Immunocompromised (sek-1)	19417±549 (n=23)	17342±599 (n=11)	11043±277 (n=35)	14107±909 (n=7)	7917±950 (n=8)	6417±852 (n=6)
	p-value	****	****	****	****	****	****
